# The Battle between Infection and Host Immune Responses of Dengue Virus and Its Implication in Dengue Disease Pathogenesis

**DOI:** 10.1155/2013/843469

**Published:** 2013-02-10

**Authors:** Peifang Sun, Tadeusz J. Kochel

**Affiliations:** ^1^Henry M. Jackson Foundation for the Advancement of Military Medicine, Bethesda, MD 20817, USA; ^2^Department of Viral and Rickettsial Diseases, Naval Medical Research Center, Silver Spring, MD 20910, USA

## Abstract

Dengue virus (DENV) is a mosquito-transmitted single stranded RNA virus belonging to genus *Flavivirus*. The virus is endemic in the tropical and subtropical countries of the world, causing diseases classified according to symptoms and severity (from mild to severe) as dengue fever, dengue hemorrhagic fever, and dengue shock syndrome. Among a variety of human cell types targeted by DENV, monocytes, macrophages, and dendritic cells are members of innate immunity, capable of mounting rapid inflammatory responses. These cells are also major antigen presenting cells, responsible for activating the adaptive immunity for long-term memory. This paper is an overview of the current understanding of the following mutually affected aspects: DENV structure, viral infectivity, cellular receptors, innate immune response, and adaptive immunity.

## 1. Introduction

Dengue virus (DENV) is an arthropod-borne single stranded RNA virus of genus *Flavivirus. *It is comprised of 4 closely related but antigenically distinct serotypes, DENV-1, -2, -3, and -4. The virus is endemic in more than 100 tropical and subtropical countries of the world. Presently no specific therapies or vaccines are available to treat diseases or to prevent DENV transmission [1, 2]. 

Illnesses caused by DENV infection include undifferentiated fever, dengue fever (DF), dengue hemorrhagic fever (DHF), and dengue shock syndrome (DSS) [3, 4]. According to WHO's 1997 documentation, DF is clinically defined as an acute febrile illness with two or more manifestations of headache, retroorbital pain, myalgia, arthralgia, rash, and so on. Symptoms of DF can last 2–7 days. DHF are defined by the following criteria: persistent high fever, hemorrhage tendency, hemoconcentration (>20%), and platelet counts (<100,000) [5–8]. DHF is further classified into 4 grades according to the severity of bleeding and plasma leakage. DSS refers to DHF grades III and IV. In many areas, severe dengue illness cases rarely present all the above 4 criteria to be defined as DHF cases. Therefore, new guidelines for classification of dengue disease severity were published by WHO in 2009; however, the 1997 case definition is still in use. Currently DHF and DSS represent 2.5 to 40% of total hospitalized dengue cases [9–11], and children are most vulnerable [12, 13]. 

Besides nutrition, age, and sex [14, 15], the following 3 factors, viral strain virulence, host genetics, and host immune status, are major contributors to DHF. Typically, dengue illnesses begin 5–7 days after a blood meal from an infected mosquito. Viremia in human peripheral blood peaks during the early days (first 2-3 days) of acute illness and then decline sharply. Higher viremia titers in DHF patients compared to that in DF cases were observed. In general, immune responses are essential for the resolution of DENV infection; however, as DHF is associated with secondary infections and symptoms of DHF emerge at the time when viremia is declined, DHF/DSS is thought to be consequences of immunopathology [16]. The three human cell types, monocytes, macrophages (MΦ), and dendritic cells (DCs), are permissible for DENV infection. These cells are major phagocytic cells of the innate immune system, responsible for detecting and removing invasive pathogens. They are also antigen presenting cells critical for the initiation, expansion, and polarization of adaptive cellular immunity. Targeting these cells by DENV may have a significant impact on immune modulation. In this paper, we summarized recent findings regarding the viral structures, host cells, and receptors, and host's innate and adaptive immune responses, for a better understanding of DHF pathogenesis. 

## 2. Dengue Virus Structure and Life Cycle

The DENV particle is coated with a host cell-derived lipid bilayer. The genome of DENV is an 11 kb-long single positive-sensed RNA molecule encoding 10 viral proteins: 3 structural proteins, capsid (C), membrane (M), envelope (E), and 7 nonstructural proteins (NS), NS-1, 2A, 2B, -3, -4A, -4B, and -5. DENV enters into host cells through receptor-mediated endocytosis. The E proteins on viral surface are major players for viral attachment, endocytosis, uncoating, and fusion. The E protein contains 3 functional domains: domain I (130–185), domain II (50–130, 185–300), and domain III (300–400). Domain II contains a fusion peptide, while domain III is considered as a binding domain that binds to cellular receptors and direct DENV particles to host cell's endosomal compartments. Viral uncoating occurs in the cellular endosomal compartments in an acidic environment, releasing viral RNA into the cytoplasm. As a positive sense (messenger) RNA, protein translation is initiated immediately after virus uncoating. The genome RNA initially produces an uninterrupted polyprotein which is subsequently cleaved into individual proteins in host cell's ER lumen and cytoplasm. The cleavage of prM, E, NS1, and NS4B proceeds those of the other NS and C. Host cell proteases and viral protease NS2B-NS3 are required for viral protein cleavage. The NS3 is also a helicase, which joins NS5, the RNA-dependent RNA polymerase, to enable viral RNA replication in the so-called viral replication complexes (RCs) near the cellular membranes in cell cytoplasm. Newly synthesized E and PrM insert into the ER membrane, whereas the newly synthesized RNA associates with C to form nucleocapsid at the cytosolic side of the ER membrane. Through a mechanism termed “budding,” the nucleocapsids join with the membrane bound PrM and E, forming progeny viral particles in the rough ER cisternae. These viral particles are transported to the Golgi apparatus and then are carried within the secretory vesicle to cell surface for extracellular release. A Golgilocalized furin protease cleaves prM at a late stage of viral replication and the mature progeny virion secreted extracellularly contains M protein. 

## 3. Viral Infection

### 3.1. Protein Glycosylation of DENV

During viral replication, protein translation in host cell's ER is continued with protein modification in ER and Golgi apparatus. One of the most common forms of protein modification is N-linked glycosylation, in which a high mannose core is attached to the amide nitrogen of asparagine (Asn) in a consensus sequence of Asn-X-Ser/Thr; X represents any amino acid. The protein modification initiates early in protein synthesis in ER and continues during protein transit through the ER to Golgi. In Golgi, the initially attached glycan is further modified by a complex process of trimming or remodeling, resulting in varying oligosaccharide structures. Glycosylation can promote proper protein folding required for protein functioning, affect interaction between virus and receptors, and alter antigenic structures recognized by host immune cells and antibody, thus impact viral replication and infectivity. 

Currently, N-linked glycosylation has been shown to affect infection, genome replication, and progeny virion packaging of DENV [17–19]. The E protein of DENV is glycosylated at position Asn67 and Asn153 [17]. While the glycosylation site at Asn-153 of E is conserved in most Flaviviruses, the site at Asn-67 is unique for DENV [17]. Other glycosylation sites are located at PrM positions 7, 31, and 52 and within NS1 at positions 130 and 207 [20]. 

Depending on the types of receptors and types of host cells studied, the E protein glycosylation affects the viral infectivity and replication differently [17]. For mammalian cells (e.g., Vero cells, BHK cells), removing glycosylation of Asn-153 by gene mutation reduces the capability of the DENV to infect host cells, whereas removing Asn-67 neither affect infectivity nor replication of the viral genome but reduces virus progeny assembly. Therefore, it is suggested that Asn-67 is important for proper folding of the newly synthesized E protein, virion assembly, and release of virions in mammalian cells. For mosquito cells (C6/36, a cell line derived from the larvae of *Aetis albopictus*), removing glycosylation at either Asn-153 or Asn-67 by gene mutation is not as crucial as for mammalian cells. Infection of C6/36 mosquito cells with either the Asn67 or Asn67/Asn153 mutants resulted in the introduction of a compensatory mutation, K64N, restoring glycosylation in the area [21].

Direct evidence regarding the change of glycan compounds on *Flavivirus* surface that may impact viral tropism is from West Nile virus (WNV). Dendritic cell-specific intercellular adhesion molecule 3-grabbing nonintegrin (DC-SIGN, CD209) and its homology liver/lymph node-specific ICAM-3 grabbing nonintegrin (L-SIGN also known as DC-SIGNR or CD209L) are expressed on different cells of different tissues and organs. DC-SIGN is expressed on dendritic and macrophages under dermal and in lymphoid tissues, whereas its homolog L-SIGN is expressed on liver sinusoidal endothelial cells. Glycosylation of E protein at position Asn-67 is critical for the interaction of DENV with the carbohydrate recognition domain (CRD) on the DC-SIGN molecule [17]. Different from DENV, WNV does not have N-linked glycosylation on Asn-67. While DENV is equally infectious for DC-SIGN and L-SIGN cells, WNV preferentially infects L-SIGN cells. The introduction of glycosylation at Asn-67 abolished this preference and rendered WNV equally infectious to both DC-SIGN and L-SIGN cells. Studies show that mannose-rich glycans on WNV were required for its interactions with DC-SIGN, but not for L-SIGN, whereas complex glycans, particularly N-acetylglucosamine terminated structures, were important for interaction with L-SIGN. This study suggested that the site of *N*-linked glycosylation on E protein molecule determines the types of glycans incorporated, thus controlling viral tropism for DC-SIGN or L-SIGN-expressing cells.

The glycan biosynthesis pathways do not need a genetic template. Glycan structures vary among species and are modulated by factors that can differ greatly among cell types. These factors contribute to the difficulty for us to understand the complicated nature of virus glycosylation. An evolving technology, glycan microarray, may be a powerful tool for profiling glycan molecules that are important for infectivity and immune recognition, thus helpful for vaccine and drug development.

### 3.2. Receptors and Host Cells Identified *In Vitro* for DENV Infection

A broad range of host cells have been documented for DENV infection *in vitro*. DENV can infect kidney-derived cells (Vero cells) from African green monkeys, baby hamster kidney cells (BHK cells), mosquito cells (C6/36), and more. Identified cellular receptors on these cells include laminin receptor [22] and other not fully characterized polypeptides [23]. In human, DENV infection has been found in monocytes, MΦ, DCs, endothelial cells, and hepatocytes [24]. Cellular receptors for hepatocytes include heat shock protein (Hsp)70 and Hsp90 [25–28], GRP78 [25], and heparin-sulfate [29]. Cellular receptors for monocytes and MΦ include mannose receptor (CD205) [30], CD14-associated protein [31], CLEC-5A [32, 33], and heparin-sulfate [29], DC-SIGN (CD209) [34]. For DCs, the cellular receptors include DC-SIGN [34]. Immature DCs express higher levels of DC-SIGN compared to mature DCs. Infectivity in immature and mature DCs differs significantly [34]. Among DCs, monocytes, and MΦ, DCs are the most permissible cells for DENV infection [35]. The high infectivity in DCs is not only attributed to higher receptor-mediated uptake, but also due to higher genome replication and *de novo* viral protein production [30]. Some suggest that DC-SIGN only serves to concentrate the virus on the cell surface; the internalization of the virus depends on another molecule since the truncated DC-SIGN lacking the endocytosis domain did not abolish virus replication [36]. 

The receptor for endothelial cells is not yet identified. The DC-SIGN homology L-SIGN [34] is thought to be the receptor for liver sinusoidal endothelial cells. For C6/36 cells, two surface proteins of 40 and 45 kDa (a putative heat shock protein) were found to interact with DENV-4 [37, 38], and a receptor of 50 kDa was found to bind to DENV-2, -3, and -4 [22], thus suggesting that multiple proteins may be used as receptors. For Vero cells, heparin sulfate and two cell surface proteins of 74 and 44 kDa mediate DENV binding [23]. According to these studies, the carbohydrate residues are important in virus binding to both C6/36 and Vero cells. Heparin sulfate is a glycosaminoglycan occurring in the cell membrane of most cells. It is assumed that heparin sulfate serves to concentrate viruses on the cell surface, and endocytosis of DENV may be dependent on another molecule. Infection through heparin sulfate has been reported for DENV-2 and -4 [39, 40]. 

### 3.3. Host Cells Identified *In Vivo* for DENV Infection

One approach that has been used to identify host cells in naturally infected humans is the histochemistry of autopsy samples from fatal dengue cases. DENV genome and immumofluorescent staining of DENV protein antigens are found mainly in phagocytic cells in lymph node, spleen, and lung [41, 42] by *in situ* RNA hybridization or immunofluorescent staining (e.g., NS-3). DENV infection was also found in perivascular cells in brain, in hepatocytes in liver, and in endothelial cells in spleen. In peripheral blood, DENV antigens were detected in CD14^+^ monocytes [43]. These studies suggested that tissue MΦ, blood monocytes, liver hepatocytes, and endothelial cells are target cells for DENV infection. Of note, DENV viremia is reported to be negative upon the time of defervescence and before the onset of DHF; therefore, the above-mentioned histochemistry studies may highlight more of a picture of late stage dengue tropism. A humanized mouse model may be useful to gain some light regarding a dynamic picture of DENV tropism [44]. This model showed that DENV first emerged (from day 1) outside the follicle-like structures (where T and B cells reside) of the spleen, and then in follicle-like structures (day 10). From day 14 to 18, DENVs were found outside the follicle areas. A similar pattern was found in bone marrow. These data suggested that non-T and non-B cells, such as DCs, MΦ, and monocytes, are targeted first by DENV. Upon migration, these cells spread DENV to T and B, and then infection goes on to other parts of the body, such as liver and lung. 

### 3.4. Receptor Usage and Viral Virulence

Receptor preference is a key for tissue tropism and virulence of the virus, and so far, little is known regarding *in vivo *receptor usage during natural DENV infection and how it affects dengue disease severity. A few animal studies may shed some light on this aspect of research. 

Adaptation of a DENV-4 isolate in DBS-FRhL-2 cells generated a variant isolate with a mutation of Glu327-Gly in E domain III. This variant virus showed increased affinity for heparin sulfate and reduced infectivity and immunogenicity in rhesus monkeys compared to the unpassaged DENV-4 [45]. Another study showed that a mouse-passaged DENV strain with reduced affinity for heparin sulfate causes severe disease in mice by establishing increased systemic viral loads [46, 47]. A recombinant virus which showed weaker affinity for heparan sulfate had an increased serum half-life, higher systemic viral loads, and high levels of TNF-*α* in the serum of infected mice [47]. It is possible that different affinities to heparin sulfate could lead viruses to different tissues where the microenvironments or cell types hosting DENV do not support optimal DENV replication or spreading. 

The role of DC-SIGN in DENV pathogenesis has been observed on genetics level. A single nucleotide polymorphism (SNP) study linked the polymorphism in the promoter region of CD209 (−336 A/G; rs4804803) with disease protection or severity [48]. The study looked at two genotypes, A/A and A/G of this promoter region and found a strong association between GG/AG genotypes of rs4804803 and risk of DHF, whereas the AA genotype was associated with protection against DENV infection [49]. The DCs generated *in vitro *from AG genotype had a higher DC-SIGN expression compared to AA genotype. However, it is puzzling that the higher DC-SIGN expression did not link to a higher level of infection; instead it linked to higher cytokine production. The TNF-*α*, IP-10, and IL-12p40 were significantly higher in DCs from AG genotype compared to AA genotype, suggesting that the innate immunity may play a critical role in disease severity.

### 3.5. Infection through Antibody-Dependent Enhancement (ADE)

One of the major risk factors for DHF/DSS is the patient's previous exposure to DENV. Primary DENV infections usually result in noncomplicated DF and the development of both humoral and cellular immunity; both are long term and protect the host from reinfection from the same serotype. Although this antidengue immune response is cross-reactive in nature, it does not confer long-term cross-protection to other serotypes. Instead, an association of DHF with secondary infections was observed [50–52], providing the immunological basis for dengue pathogenesis. 

The theory known as ADE suggests that the presence of nonneutralizing serum Abs from a previous exposure mediate an enhancement of infection to subsequent heterologous DENV infections. The process of ADE involves the binding of Ab to DENV, forming Ab-DENV immune complexes (ICs), and the binding of the complexes to FcR, resulting in an increased DENV uptake by FcR-bearing cells. Investigation of ADE using *in vitro* models showed that ADE occurs in a variety of primary cell cultures and cell lines, including human plasmacytoid DCs, mature DCs, and monocytes. All cell types identified from *in vivo* histology as dengue target cells bear FcR, and all of them support ADE in *in vitro* experiments. Using monocytes as an example, infection of DENV in the absence of immune sera was less than 1%, but in the presence of sera Abs, infection can increase to >10-fold [53]. To address the role of ADE in dengue disease pathogenesis, sera from the subjects living in the endemic regions of the world who were enrolled in prospective cohort studies were evaluated in *in vitro *ADE assays. The ADE titers in preillness sera did not correlate with the clinical severity or viral burden of secondary DENV infection [54], suggesting other factors are important in the pathogenesis of DENV infection. Some of these factors could be the innate immune responses triggered by ICs of Ab-DENV. 

## 4. Innate Immune Responses

### 4.1. Immune Complexes (IC) Triggered Innate Immunity

The majority of ICs formed between Abs and pathogens are cleared from the circulation in the liver and spleen by MΦ. The IC can be phagocytosed through binding to FcR expressed by phagocytic cells, and the result is the degradation of the pathogens by enzymatic activities in the lysosomal compartments. Secondarily, foreign antigens expressed on the cell surface (e.g., Ab-DENV bound to FcR on cell surface, NS-1 expressed by infected cells) can be recognized by NK cells, triggering a killing mechanism known as Ab-dependent cellular cytotoxicity (ADCC) which results in the killing of infected cells [55]. Further, IC can activate the complement system which damages the infected cells, restricting virus propagation in infected cells [56]. All of these mechanisms of innate immunity are activated immediately upon pathogen invasion and play important roles in controlling pathogenic infection. At the meantime, the killing of target cells is associated with inflammatory cytokine/chemokine responses [57–59]. 

The effect of ADCC with respect to its protective role against DENV secondary infection has been documented. A study evaluated the level of ADCC in a ^51^Cr-release assay using preexisting DENV-positive plasma obtained prior to heterologous secondary DENV-2 and -3 infections through a prospective cohort study of the Thai school children. The principal of this study is the IC formed on the infected cell surface (plasma Abs bind to DENV antigens expressed by infected cells) are recognized by the FcR (CD16) on the NK cells, leading to NK cell killing of DENV-infected cells. Results showed that higher ADCC activities associated with higher plasma neutralizing Ab activities. Higher ADCC activity in presecondary DENV3 infection plasma samples correlated with lower plasma viremia levels, although this correlation was not seen with presecondary DENV2 infection plasma samples. No overt association was seen between ADCC activity and the clinical outcomes of disease severity in secondary infections, but the lowest ADCC activities were found to correlate with DHF of DENV-3 secondary infections. ADCC may contribute to the early control of secondary DV3 viremia *in vivo* [60]. 

DENV IC can activate complement pathway. A study used a total of 33 E-specific MAbs against DENV2 and 43 against DENV4 to study ADE showed that all MAbs enhanced infection at subneutralizing doses under normal ADE assay conditions where test samples were heat inactivated. However, the inclusion of commercial rabbit complement or fresh sera from healthy humans in the ADE assay system abolished the ADE activities of all these MAbs. Complement C1q- or C3-depleted sera had a little effect on the elimination of ADE. Fresh human sera tended to eliminate ADE more effectively in homologous than heterologous viruses [61]. The complement component C1q restricts ADE by anti-*Flavivirus* Abs in an IgG subclass-specific manner in cell cultures and mice. IgG subclasses that bind C1q avidly induce minimal ADE in the presence of C1q, whereas subclasses that bind C1q weakly enhance infection strongly [62]. On the other hand, the complement system is activated in DHF/DSS. The peak of activation and the presence of C3a and C5a anaphylatoxins coincided with the onset of shock and leakage. The levels of C3a correlated well with disease severity. This indicated an important role of the complement system in the pathogenesis of shock [63].

Innate immunity triggered by the Ab-DENV IC may play a role in dengue disease severity. There are 4 isotypes of IgG in human serum: IgG1 makes up most of (65%) the total IgG in human serum, followed by IgG2 and then IgG3 and IgG4. The amounts of serum IgG3 and IgG4 are similar. IgG1 usually binds to FcR with greater affinity than IgG2. There are three major subclasses of FcR: Fc*γ*RI (CD64), expressed on monocytes, MΦ, neutrophils, myeloid precursors, and DCs; Fc*γ*RII, the most widely distributed human Fc*γ*R type, expressed on most types of blood leukocytes, DCs, and platelets; and Fc*γ*RIII (CD16) is expressed on NK cells and MΦ [47]. Fc*γ*R I has high affinity for monomeric human IgG1 and IgG3 and low affinity for IgG4; is not binding to IgG2. Fc*γ*RII is a low-affinity receptor which only binds aggregated IgG. It is the only Fc*γ*R class able to bind IgG2. 

Due to structural characteristics of Ig isotypes and FcgR, the functional property of each Ig isotype is different. IgG1 and IgG3 can fix complement much more effectively than that of IgG2 [2, 5]. Kinetics and levels of lgG1–4 against each DENV serotype from patients with DF, DHF, and DSS have been studied. IgG1 and IgG3 serum Abs were the predominant Ig throughout the course of illness in all patients. Serum levels of IgG1 and IgG3 are significantly higher in DHF, and DSS patients than in DF patients [64, 65]. In opposite, IgG2 are significantly lower in DHF and DSS patients than in DF patients [64]. Significant difference of IgG4 was also found between the DHF/DSS and DF patients [65]. The role FcR polymorphism in dengue pathogenesis is also being explored. Fc*γ*R II A has two codominantly expressed alleles R131 and H131, which differs at 2 amino acids at positions 27 and 131. R131 and H131 differ significantly in binding to IgG2 and IgG3. Low binding homozygotes R131 of the Fc*γ*R IIa are implied in protection from DHF [66]. 

Strategies to reduce IgG-Fc*γ*R binding to minimize ADE were explored by altering the Ab Fc structures responsible for binding to Fc receptors. IgG 1A5 variants, containing amino acid substitutions from the Fc region of IgG2 or IgG4 antibodies, reduced but did not eliminate DENV-4-enhancing activity in K562 cells. Importantly, a 9-aa deletion at the N terminus of the CH_2_ domain in the Fc region abrogated the enhancing activity [67]. 

These studies suggested that Ab-neutralization and ADE of DENV are more complicated events. It is a balance of many factors: the concentration and specificity of the Ab, the Ig isotypes of the Ab, the receptor and host cell type, the complement and ADCC, and so forth. The fact that the addition of complement in a conventional plaque reduction neutralization test (PRNT) can turn nonneutralizing Ab to neutralize viral infection suggests that nonoptimal Abs depend on complement and the innate immune system to control viremia. The activation of ADCC and complement fixation may occur immediately upon viral infection, resulting in a strong inflammatory response, for example, NK cell and macrophages activation and inflammatory cytokine/chemokine production, thus predisposing patients with more cell/tissue damage. 

The current widely used approaches, such as PRNT or ADE assays, addressed only partial functionality of DENV immune sera. Future efforts should be made to understand the contribution of innate immune activities triggered by DENV-Ab IC to the Ab-neutralization/enhancement, and to disease pathogenesis.

### 4.2. DENV Infection and Cell Maturation/Activation and Cytokine Production

DCs, monocytes, and MΦ are groups of heterogeneous bone-marrow-derived cells that are classified as important members of the innate immune system. DENV infection of these cells induces cytokine production and cell activation and maturation. 

The production of IL-6, IL-8, IP-10, and TNF-*α* in DENV-infected monocytes was found to correlate with maximum virus production. DENV infection through the mechanism of ADE shows to induce the production of IFN-*α*, TNF-*α*, and IL-10 and upregulation of costimulatory markers CD40 and CD86, in primary *in vitro* monocyte cultures [53]. *In vivo* [68], the numbers of CD14^+^ monocytes expressing the adhesion molecule intercellular adhesion molecule 1 (ICAM-1), TLR2, TLR4, and CD16 were increased during the acute stage of DF. The two major types of blood monocytes, CD14^++^ CD16^−^ and the CD14^+^ CD16^+^, show distinct phenotype and function: CD14^+^ CD16^+^ are proinflammatory and have a higher expression of proinflammatory cytokines and higher potency in antigen presentation, and they rise in numbers in many disease processes [69]. It is not clear why this subset is increased in DF patients but not in DHF patients. 

In humans, two major lineages of human dendritic cells have been studied extensively: myeloid DC (Lin^−^CD11c^+^CD123^med^) and plasmacytoid DC (Lin^−^CD11c^−^CD123^high^). Myeloid DCs are distributed in various tissues that provide an environmental interface, such as skin (Langerhans cells), mucosal tissues of nose, lung, stomach, and intestine, where they filter antigens and become sensitized. It is suggested that dermal DCs are primary target cells when DENV is first injected into the skin by a mosquito bite. Most of the DC studies relating to DENV were carried out using cells derived from blood monocytes *in vitro* under the influence of IL-4 and GM-CSF [35, 70–72] or cells migrated from explanted skin patches in culture [73, 74]. These studies showed that DENV-infected DCs produce inflammatory cytokines, TNF-*α*, IFN-*α*, IL-6, regulatory cytokine IL-10, and chemokines IFN-*γ*-inducible chemokines CXCL9, 10, 11; IL-12p70 is not produced by DENV-infected DCs unless costimulatory ligand or inflammatory cytokine (IFN-*γ*) are present [72, 75]. DENV is capable of impairing DC maturation and suppress T-cell proliferation [75, 76]. Infection of plasmacytoid DCs by DENV is not as apparent as myeloid DCs, because the infection rate (percent of infected cells in a culture) determined by MAb-staining is very low. However, DENV replication in plasmacytoid DCs is confirmed by confocal microscope [77] and by the detection of negative stranded RNA [34]. Vigorous production of type-I IFN (IFN-*α*) was seen in DENV-infected plasmacytoid DC cultures. 

The ability of DENV-infected MΦ to induce permeability changes in primary human HUVEC was investigated. Supernatants from DENV-2-infected MΦ increased permeability in HUVEC monolayers without infecting HUVEC cells. Although permeability induction was enhanced by pre-incubation with supernatants from infected MΦ harvested at the time of peak release of TNF-*α* and infectious virus, TNF-*α* does not seem to be responsible for HUVEC permeability in this study. Nevertheless, this model system can be used for further *in vitro* analysis of mechanisms that may relate to capillary leakage and the development of DHF/DSS [78]. 

TLRs are pattern recognition receptors employed by the innate immune system to recognize pathogen-associated molecular patterns broadly shared by groups of microbes. Among 11 TLRs identified so far, three TLRs, TLR3, 7, and 8, are important for sensing invasion of RNA viruses. TLR3 recognizes double stranded RNA, whereas TLR7 and 8 recognize single stranded RNA. The downstream signaling events of TLRs activate the type I IFN (IFN-*α*/*β*) transcription factors and the production of IFNs. DENV is a single stranded RNA virus, and its replication relies on the formation of double stranded RNA intermediates. It is shown that TLR7 is required to recognize DENV in plasmacytoid DCs [70] for type I IFN production. TLR3 is responsible for recognition of DENV and triggering cytokine production in human monocytic cell line U937. Colocalization of TLR3 and DENV RNA upon DENV internalization was observed. TLR3 can mediate strong IFN-*α*/*β* release to inhibit DENV replication, thus limit the cytopathic effect [79]. TLR2 and TLR4, which recognize mostly glycan-lipids, were also suggested in the activation of monocytes upon DENV infection and inflammatory cytokine production [68]. 

Apoptosis and cytokine profiles have been used in *in vitro *models for assessing virulence of DENV isolates. It seemed that more virulent DENV isolates cause more extensive cell apoptosis [80] and induce higher amounts of cytokine/chemokine production. Infection of human monocyte-derived DC with a clinical isolates from a nonfatal case of DF from Brazil in 2002 and a fatal case with visceral complications from Paraguay in 2007 showed that the strain from fatal case display significantly higher replicative ability than that of the nonfatal case. In addition, the strain of fatal case elicited increased the production of proinflammatory cytokines and higher rates of cell apoptosis [81]. In consistency with these observations, higher serum levels of inflammatory cytokines/chemokines (TNF-*α*, IFN-*α*, IL-1, IL-6, IL-8, IL-10, etc.) have been found in DHF patients versus DF patients. Therefore, apoptosis, cell activation, and cytokine production may directly contribute to the clinical manifestation of dengue diseases.

### 4.3. Apoptosis of DENV-Infected Cells

Apoptosis is documented in almost every single type of cells infected by DENV *in vitro* [76, 82] and *in vivo*. *In vitro*, apoptosis was documented in DENV-infected DCs, monocytes/MΦ, hepatocytes, endothelial cells, and so forth. Apoptosis of infected monocytes was found hours after infection with nuclear condensation and fragmentation, cellular shrinkage, blebbing, and budding. Nuclear DNA degradation was confirmed by TdT-mediated dUTP nick-end labeling (TUNEL) technique [83]. *In vivo*, apoptosis was studied by colabeling the cells with TUNEL agents and DENV antigens in an immunohistochemical assay. During a DENV-2 outbreak in Santiago de Cuba in 1997, apoptotic cells were found in five of the six fatal cases studied. Apoptosis was demonstrated in liver, brain, and intestinal and lung tissues, in cerebral cells, white blood cells, intestinal and pulmonary microvascular endothelial cells (ECs) [84]. It is speculated that the apoptosis of microvascular ECs in pulmonary and intestinal tissues is related to vascular plasma leakage.

Apoptosis is a mechanism of cell death involved in the regulation of tissue homeostasis. The two major pathways of apoptosis are the extrinsic (death-receptor dependent signaling pathway) and the intrinsic (mitochondria-associated) pathways, both of which are found in the cytoplasm. The death-receptor dependent signaling pathway (also known as the TNFR-mediated apoptosis pathway) [85] involves death receptors such as Fas, DR3, TNFR-1 and -2, DR4, DR5, DR6, and so forth. The ligands are FasL for Fas, TNF-*α* for TNFR-1 and -2, TNF-related apoptosis-inducing ligand (TRAIL), and so forth. Apoptosis is initiated by binding the ligand to receptor, clustering the receptors on the cell surface, triggering a stream of intracellular signaling events involving FADD (Fas-associating protein with death domain), DISC (death-inducing signaling complex), and a set of caspases, leading to the degradation of cellular proteins necessary to maintain cell survival and integrity. The intrinsic pathway occurs when various apoptotic stimuli trigger the release of cytochrome C from the mitochondria (independently of caspase-8 activation). Cytochrome C interacts with Apaf-1 and caspase-9 to promote the activation of caspase-3. Recent studies point to the ER as a third organelle implicated in apoptosis. The primary function of the ER is to facilitate protein folding and secretion. A number of stress conditions can lead to accumulation of unfolded and/or misfolded proteins, which interfere with ER's function, a condition termed “ER stress.” As a result, cells activate an integrated intracellular signaling cascade, the UPR, to avert ER stress. However, prolonged ER stress can activate cell apoptosis. 

The pathways that lead to the apoptosis of DENV-infected cells have been studied *in vitro*. Results indicated that a combination of these pathways may work together. DENV-induced apoptosis mediated by the unfolded protein response (UPR) has been evaluated A549 cells. Upon DENV infection, A549 cells elicit an UPR which is observed at the level of translation attenuation (as visualized by the phosphorylation of eIF2alpha) and activation of specific pathways such as nuclear translocation of ATF-6 and splicing of XBP-1. Modulators of UPR can inhibit DENV replication [86]. The death-receptor dependent signaling pathway was found to be involved in DENV-infected hepatic cell apoptosis [87]. Death-receptor dependent apoptosis pathway is also implicated in apoptosis of DENV-infected HUVEC cells [88]. The expression of cell death genes including RIPK2, HRK, TGF-beta, PERK, and LC3B during DENV-infection of HepG2 cells is consistent with the activation of apoptosis and autophagy [89]. RIPK2 belongs to the receptor-interacting protein family of serine/threonine protein kinases, which is a crucial mediator of multiple stress responses that leads to the activation of caspase, NF-kappaB, and MAP kinases including JNK and p38. 

DENV C protein, NS3 protease (NS3pro), and NS2B-NS3 serine protease precursor protein (NS2B-NS3(185)(pro)) are implicated in apoptosis. The C protein physically interacts with the human death domain-associated protein Daxx. A double substitution mutation in DENV C (R85A/K86A) abrogates Daxx interaction, nuclear localization, and apoptosis. Expression of CD137, a member of the TNF receptor family, increased significantly in HepG2 cells expressing DENV C compared to HepG2 cells expressing DENV C (R85A/K86A) [90]. Human microvascular endothelial cells (HMEC-1) infected with a DENV-2 clinical isolate, or HMEC-1 cells transfected with NS3pro or NS2BNS3pro were able to trigger apoptosis after 24 h of infection or transfection: cytoplasmic shrinkage, plasma membrane blebbing, TUNEL positivity, caspase-3 activation, and cleaved PARP, a central regulator of apoptosis [91]. Site-directed mutagenesis which replaced His(51) with Ala within the protease catalytic triad significantly weakens the NS3- and NS2B-NS3(185)(pro)-induced cell apoptosis [92].

Apoptosis of peripheral blood mononuclear cells (PBMCs) was examined in cohorts of DF and DHF children. Around defervescence, PBMC apoptosis was higher in children with DHF, compared to DF and nondengue febrile cases. CD8^+^ T-lymphocytes comprised at least half of the peak apoptotic PBMC in children. Apoptosis was also found in DENV peptide-specific CD8^+^ T cells from patients with acute illness. One possible mechanism of T-cell apoptosis is the apoptosis of DENV-infected antigen presenting cells (dendritic cells, macrophages, etc.) which caused T-cell apoptosis. 

It is common for a virus to infect a cell and trigger its programmed cell death pathway. Apoptosis is considered a host defense mechanism. Sudden cell death triggers the immune system to remove cells harboring harmful pathogens. However, it may also maximize viral spread from lytic cells. Many viruses have been shown to trigger apoptotic cell death and/or encode inhibitors of apoptosis. Although these viral factors have been studied in great molecular detail, it is less clear how these factors contribute to innate defense or disease pathogenesis. 

## 5. T-Cell Responses to DENV

Cell mediated immunity is comprised of two major subsets of the T cells, CD4 and CD8. CD4^+^ T cells exert functions as helpers for other T cells and B cells, whereas CD8^+^ exerts cytotoxic function. CD4^+^ T cells can be divided into Th1 and Th2 subtypes based on their cytokine profiles. Th1 cells produce IFN-*γ*, TNF-*α*, and IL-2; Th2 cells produce IL-4, IL-5, IL-10, and IL-13. The IFN-*γ* and TNF-*α* can have a direct killing effect on intracellular pathogens while IL-2 is required for helping with T-cell proliferation. Therefore, Th1 response is important for antibacterial and antiviral immune defense. CD8^+^ T cells can directly recognize and kill infected cells through cytotoxicity (CTL) and antiviral cytokines such as IFN-*γ*. Recently, it was shown that a strong polyfunctional CD8^+^ T cell response capable of coproducing TNF-*α* and IL-2 in additional to IFN-*γ* [93–95] was required to control the progressive infection of viruses like HIV and HCV. 

The major target cells for DENV, monocytes, MΦ, and DC are APCs critical for stimulating cell mediated immunity. Targeting these cells by DENV may have an unfavorable impact on host adaptive immunity. *In vitro*, infected DCs promote IFN-*γ* production from T cells [71, 72, 75, 82, 96]. However, the response is altered. It is shown that DENV-infected DCs are incapable to prime a mixed lymphocytes reaction (MLR) [82]. Others showed that DENV-infected DCs induced initial proliferation of naive CD4^+^ T cells, but they remained nonpolarized in effector function. The expression of IFN-*α*/*β*-stimulated genes was downregulated [97]. In naturally infected humans during acute disease stages, it seems that cellular immunity is not fully activated and apoptosis is observed based on the following observations: impairment of CD8^+^ T-cell cytokine production [98], decreased circulating of CD4^+^ and CD8^+^ T-cell counts [99], impairment of T-cell proliferation [100, 101], and increased T-cell apoptosis. The *in vitro* studies on DENV-infected DCs supported these observations showing that DC maturation was abolished and apoptosis was observed, and T-cell proliferation was significantly suppressed [75, 82]. 

*In vivo*, the T-cell activities, T-cell counts, and cytokine production are all restored after the early illness (day 5 and on) at the time when viremia declines [98–101]. Often, the amount of DENV-specific T cells, measured by cytokine assays and tetramer staining, recovered from DHF patients are higher than that of DF patients; therefore, cellular immunity is currently considered to play a pathological role [102–104]. By examining the intracellular cytokine profile, the ratio of IFN-*γ*/IL-4 and the percentage of Mip-1^+^ CD8 T cells are found higher in DHF patients than in DF patients [105], suggesting a Th1-type of memory cell response. T-cell responses in a cohort of dengue-infected children from Thailand are found to target to most of the 10 viral proteins. However, responses to NS3 is the most dominant, and there is a very strong association between the magnitude of the response to NS3 with disease severity [102]. Specifically, the cross-reactive memory T cells recovered from dengue illness exhibited higher affinity to variant epitopes representing serotypes of previous exposure—a phenomenon termed “original antigenic sin” [98]. 

A major limitation for understanding DENV-specific immune responses is associated with the study design in those studies that have used samples taken from patients in the acute phase of their illness through their recovery period rather than samples obtained prior to the infectious process [98, 101, 102, 106, 107]. More importantly, those studies did not include asymptomatic secondary infections; therefore, they did not address the role of cellular immunity in immune protection; rather, they emphasized a correlation between antidengue cellular immunity with disease severity. An unbiased natural infection model that includes preinfection samples from both asymptomatic and symptomatic infections is needed to correct this bias. This can be accomplished through a longitudinal cohort design where scheduled blood collection is carried out in a human cohort that is monitored for disease and seroconversions. To our knowledge, only two studies published by the same group described preexisting cellular immunity in subclinical infections [108, 109]. Reyes-Del Valle et al. reported a higher proportion of IFN-*γ* and IL-2 responses to DENV-3 antigen among persons with subclinical infections compared to those with symptomatic infections. The study suggested that IL-2 maybe an important cytokine for immune protection [26].

## 6. Summary and Future Prospective

Significant progresses have been made regarding viral and host cellular molecules involved in DENV-receptor interaction and infection. However, the dynamic process of DENV tropism during different stages of DENV infection, the preference of receptors and host cells involved in the primary or secondary, and in early or late stages of DENV infections, are not clearly understood. Most importantly, the role of glycosylation with respect to receptor binding, viral tropism, and virulence of infectivity is not well understood. Current Ab-mediated neutralization/enhancement studies rarely include innate immune mechanism, such as complement activation and ADCC. Future studies should target into these challenging areas. Further, future studies on adaptive humoral and cellular immunity should use a unique population: those who experienced DENV asymptomatic infections, to better address the protective immunity.
